# The levels of soluble cMET ectodomain in the blood of patients with ovarian cancer are an independent prognostic biomarker

**DOI:** 10.1002/1878-0261.12939

**Published:** 2021-04-07

**Authors:** Daniel Martin Klotz, Theresa Link, Maren Goeckenjan, Pauline Wimberger, Jan Dominik Kuhlmann

**Affiliations:** ^1^ Department of Gynecology and Obstetrics Medical Faculty and University Hospital Carl Gustav Carus Technische Universität Dresden Germany; ^2^ National Center for Tumour Diseases (NCT) Dresden Germany; ^3^ German Cancer Research Center (DKFZ) Heidelberg Germany; ^4^ Faculty of Medicine and University Hospital Carl Gustav Carus Technische Universität Dresden Germany; ^5^ Helmholtz‐Zentrum Dresden‐Rossendorf (HZDR) Germany; ^6^ German Cancer Consortium (DKTK) Dresden Germany

**Keywords:** biomarker, ovarian cancer, prognosis, soluble cMET, therapy monitoring

## Abstract

The tyrosine kinase mesenchymal–epithelial transition (cMET) is typically overexpressed in up to 75% of patients with ovarian cancer, and cMET overexpression has been associated with poor prognosis. The proteolytic release of the soluble cMET (sMET) ectodomain by metalloproteases, a process called ectodomain shedding, reflects the malignant potential of tumour cells. sMET can be detected in the human circulation and has been proposed as biomarker in several cancers. However, the clinical relevance of sMET in ovarian cancer as blood‐based biomarker is unknown and was therefore investigated in this study. sMET levels were determined by enzyme‐linked immunosorbent assay in a set of 432 serum samples from 85 healthy controls and 86 patients with ovarian cancer (87% FIGO III/IV). Samples were collected at primary diagnosis, at four longitudinal follow‐up time points during the course of treatment and at disease recurrence. Although there was no significant difference between median sMET levels at primary diagnosis of ovarian cancer *vs*. healthy controls, increased sMET levels at primary diagnosis were an independent predictor of shorter PFS (HR = 0.354, 95% CI: 0.130–0.968, *P* = 0.043) and shorter OS (HR = 0.217, 95% CI: 0.064–0.734, *P* = 0.014). In the follow‐up samples, sMET levels were prognostically most informative after the first three cycles of chemotherapy, with high sMET levels being an independent predictor of shorter PFS (HR = 0.245, 95% CI: 0.100–0.602, *P* = 0.002). This is the first study to suggest that sMET levels in the blood can be used as an independent prognostic biomarker for ovarian cancer. Patients at high risk of recurrence and with poor prognosis, as identified based on sMET levels in the blood, could potentially benefit from cMET‐directed therapies or other targeted regimes, such as PARP inhibitors or immunotherapy.

## Graphical abstract

This study suggests sMET as an independent prognostic biomarker for ovarian cancer patients at primary diagnosis and in the course of platinum‐based chemotherapy. sMET is an easily detectable serum parameter and could possibly be implemented into routine diagnostics as a complementary tumour marker for individualized prognosis stratification and for monitoring treatment response in patients with ovarian cancer.

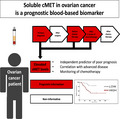

**Abbreviations**
ADAM: a disintegrin and metalloprotease
AUC: area under the curve
BMI: body mass index
CI: confidence interval
cMET: mesenchymal–epithelial transition tyrosine kinase
DDC: 3,5‐diethoxycarbonyl‐1,4‐dihydroxycollidine
ED: estimated difference
EGFR‐TKI: epidermal growth factor receptor tyrosine kinase inhibitor
FIGO: Fédération Internationale de Gynécologie et d'Obstétrique
HGF: hepatocyte growth factor
HR: hazard ratio
MMP9: matrix metalloprotease 9
NSCLC: non‐small‐cell lung cancer
OS: overall survival
PFS: progression‐free survival
ROC: receiver operating characteristic
sMET: a soluble cMET ectodomain
TPR: translocated promoter region; PARPi, Poly‐ADP‐Ribose‐Polymerase inhibitor

## 1 Introduction

Epithelial ovarian cancer is the leading cause of death among patients with gynaecological malignancies. Ovarian cancer is typically diagnosed at late stage, particularly the most common subtype of high‐grade serous ovarian cancer, with more than 70% of patients suffering of advanced disease at primary diagnosis [1]. Standard therapy for advanced ovarian cancer includes surgical debulking aimed at macroscopic complete tumour resection and platinum‐ and paclitaxel‐based chemotherapy, which prolongs progression‐free survival (PFS) and overall survival (OS) [2, 3, 4]. The postoperative residual tumour burden is the one of the most important prognostic factors in advanced ovarian cancer at primary diagnosis [1, 5]. Despite improved radical surgical debulking and the addition of novel targeted therapies to standard treatment, such as bevacizumab or Poly‐ADP‐Ribose‐Polymerase inhibitors (PARPi), the majority of ovarian cancer patients have a poor overall prognosis [6, 7, 8, 9]. Considering this clinical challenge, the identification of blood‐based predictive and/or prognostic biomarkers is of high clinical importance.

The proto‐oncogene mesenchymal–epithelial transition (*MET*) was discovered in 1984 and encodes for the receptor tyrosine kinase cMET [10]. The *MET* oncogene can be activated by the translocated promoter region (TPR), which translocates from chromosome 1 to the region upstream of the *MET* gene, resulting in constitutive activity of the cMET kinase [10, 11]. The pleiotropic protein hepatocyte growth factor (HGF) is the only known ligand of cMET to date [12, 13]. The HGF/cMET pathway controls a variety of cellular functions, such as proliferation, angiogenesis and migration, and has been associated with the metastatic progression of human cancer [14, 15, 16, 17, 18, 19]. In ovarian cancer, the HGF/cMET pathway is aberrantly activated and contributes to matrix metalloprotease 9 (MMP9)‐mediated invasion and malignant progression [20]. Overexpression of cMET was observed in up to 75% of ovarian cancer patients and was associated with poor prognosis [21, 22]. In preclinical *in vivo* models, it was shown that genetically targeting cMET reduces tumour burden and inhibits peritoneal dissemination and invasion of ovarian cancer cells through an α_5_β_1_ integrin‐dependent mechanism [23]. Pharmacological targeting of cMET signalling has already been translated into clinical trials for ovarian cancer, showing clinical activity of the cMET inhibitors cabozantinib and the monoclonal antibody rilotumumab [24, 25]. However, there was no OS benefit compared with standard treatment, possibly due to the lack of an appropriate predictive biomarker for cMET‐targeted therapy, as expression of cMET in the primary tumour was not determined in these clinical trials.

The proteolytic release of transmembrane proteins by metalloproteinases, a process called ectodomain shedding, is of fundamental biological relevance and has been observed for a variety of transmembrane proteins, including cMET [26]. It was reported that cMET overexpression results in increased ectodomain shedding of cMET, and the shedding rate of tumour cells reflects their malignant potential [27, 28]. Shedding of cMET results in a soluble cMET ectodomain (sMET) and an intracellular fragment. The former can be stably detected in the blood of healthy and diseased individuals; the latter is rapidly degraded by the proteasome [26, 27, 29, 30, 31, 32]. For several cancers, such as prostate or lung cancer, it was reported that sMET in serum or plasma reflects cMET (over)expression in the corresponding primary tumour [31, 33, 34]. Furthermore, sMET has been suggested to be a diagnostic and prognostic blood‐based biomarker in several cancers, such as uveal melanoma [35]. It was also described as a dynamic monitoring marker of epidermal growth factor receptor tyrosine kinase inhibitor (EGFR‐TKI) treatment in advanced non‐small‐cell lung cancer (NSCLC) [36]. However, the potential role and clinical use of sMET as a potential blood‐based biomarker for ovarian cancer is completely unknown.

Therefore, the objective of the current study was to investigate clinical relevance of sMET and to particularly address the question whether longitudinal sMET levels may serve as a predictive and/or prognostic blood‐based biomarker for ovarian cancer.

## 2 Patients and methods

### 2.1 Patient characteristics

The present retrospective cohort study was conducted at the Department of Gynecology and Obstetrics at the Carl Gustav Carus University of Dresden, Technische Universität Dresden, Dresden, Germany. In total, 86 patients with histologically confirmed primary epithelial ovarian cancer and primary diagnoses between 2013 and 2019 were included. Written informed consent was obtained from all study participants, as approved by the Local Research Ethics Committee in Dresden (EK74032013), and the study was performed, according to the Declaration of Helsinki. The patients' clinical data are reported in Table 1. Tumours were classified in line with the WHO‐classification of tumours derived from female genital tract, and tumour staging was classified according to the Fédération Internationale de Gynécologie et d'Obstétrique (FIGO) [37], which was revised in 2014 [38]. The latter was used for all patients who underwent surgery from 2014 onwards. All patients received radical surgery aiming at macroscopic complete tumour resection and the recommendation of platinum‐ and paclitaxel‐based chemotherapy in line with national guidelines. In case of no contraindications, patients with a tumour stage of at least FIGO IIIb were additionally treated with the monoclonal antibody bevacizumab. Primary platinum resistance was defined as disease progression within 6 months after platinum‐based chemotherapy and primary platinum sensitivity as disease progression later than 6 months of platinum‐based chemotherapy.

**Table 1:** Patient characteristics (total cohort).

*N*	86
Age	Median 62 years (37–83 years)
BMI	Median 25.9 (19.6–39.7)
FIGO	
I–II	13 (15.1%)
III–IV	73 (84.9%)
Histologic type	
Serous	75 (87.2%)
Other	11 (12.8%)
Residual tumour	
Macroscopic complete resection	42 (48.8%)
Any residual tumour	44 (51.2%)
Recurrence	
PFS	Median 17 months (1–81 months)
No relapse	34 (39.5%)
Relapse	52 (60.5%)
Survival	
OS	Median 31 months (1–81 months)
Alive	52 (60.5%)
Dead	34 (39.5%)

### 2.2 Healthy controls

In total, 85 female healthy individuals without any history of benign or malignant disease were recruited as controls. Informed written consent was obtained from all participants and approved by the Ethics committee as above (EK74032013). Control sample acquisition was performed, according to the Declaration of Helsinki.

### 2.3 Serum preparation

Serum (control and patient) sample was processed as previously described [39]. After blood withdrawal (7.5 mL S‐Monovette®, Sarstedt AG & Co., Nuembrecht, Germany), blood samples were incubated at room temperature for at least 30 min for complete blood coagulation. Within 1 h of blood drawing, serum was prepared by centrifugation for 8 min at 1800 ***g*** at room temperature and immediately frozen at −80 °C until further processing. Unnecessary freeze–thaw cycles were strictly avoided. Samples were thawed on ice and immediately processed after complete thawing. Sample identities were blinded so that time of blood drawing could not be disclosed during the analysis.

### 2.4 Detection of sMET

sMET concentrations were determined with the enzyme‐linked immunosorbent assay kit (soluble) cMet ELISA Kit (Thermo Fisher, Waltham, MA, USA), and the assays were performed according to manufacturer's instructions. The final optical readout was conducted with the microplate reader Infinite M200 and software magellan version 7.2 (Tecan, Männedorf, Switzerland).

### 2.5 Statistical analysis

The statistical analysis was conducted with r (R‐Studio version 3.6.2, R‐Studio Inc., Boston, MA, USA) and graphpad prism (GraphPad Software version 8.4.3, La Jolla, CA, USA) with statistical workflows adapted from our previous studies [39, 40]. *P*‐values < 0.05 were considered statistically significant. Confidence intervals (CI) were reported as 95% CI. Overall survival (OS) and progression‐free survival (PFS) were assessed as the time of death, last follow‐up, or disease progression from the point of primary diagnosis and assessed as separate outcome variables for all models. Nonparametric two‐sided Mann–Whitney *U*‐test was used to compare sMET levels. The Hodges–Lehmann estimate was used to determine the estimated differences in medians. Uni‐ and multivariate Cox proportional hazards regression model analyses were performed to study the prognostic relevance of sMET levels, and hazard ratios (HRs) with 95% CI are indicated. Kaplan–Meier analyses were performed with significance levels indicated with log‐rank (Mantel–Cox) analysis, and HRs (Mantel–Haenszel) are shown with 95% CI. The correlation of reported parameters was assessed by nonparametric Spearman correlation and linear regression shown. The cut‐offs for stratifying the patients into either a sMET high or low group were defined using maximally selected rank statistics, using conditional Monte–Carlo for *P*‐value approximations (Fig. S1). In order to confirm the usefulness of this unbiased method, applied herein, we exemplarily performed Kaplan–Meier analysis with an additional (arbitrarily selected) cut‐off, which was very close to the calculated cut‐off at primary diagnosis, showing no statistical significance for PFS or OS (Fig. S2). The general linear model analysis was used to analyse whether sMET levels predict primary platinum resistance. Odds ratio (OR) are reported with 95% CI. The median was used as the cut‐off for stratifying the patients into area under the curve (AUC) high or AUC low and after disease recurrence. By receiver operating characteristic (ROC) curve analysis, we assessed the ability of sMET concentrations to separate between ovarian cancer patients (total cohort or FIGO I‐II only) and healthy controls.

## 3 Results

### 3.1 sMET in ovarian cancer patients at primary diagnosis and in the course of treatment

**Fig. 1:**
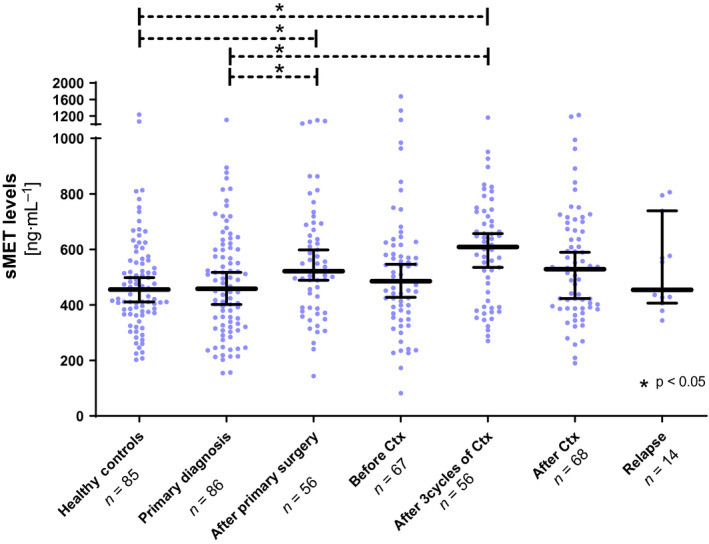
sMET levels among longitudinal sampling in ovarian cancer patients. Scatter plot showing sMET levels in healthy controls (*n* = 85), in ovarian cancer patients at primary diagnosis (*n* = 86), one week after primary surgery (*n* = 56), before platinum‐based chemotherapy (*n* = 67), after three cycles of chemotherapy (*n* = 56), after completion of chemotherapy (After Ctx, *n* = 68) and at disease relapse (*n* = 14). The black horizontal lines indicate the median sMET level in each group, with error bars showing the 95% confidence interval. *P*‐values correspond to the nonparametric two‐tailed Mann–Whitney *U*‐test for independent samples.

We analysed sMET levels in a cohort of clinically documented ovarian cancer patients (*n* = 86) and compared it to healthy controls (*n* = 85). At primary diagnosis, there was no significant difference in sMET levels between ovarian cancer patients and healthy controls (Fig. 1). Accordingly, ROC curve analysis showed that sMET does not discriminate well between healthy controls and ovarian cancer patients at primary diagnosis with an AUC of 0.510 (95% CI: 0.422–0.598, *P* = 0.82) in the total cohort and an AUC of 0.636 (95% CI: 0.451–0.821, *P* = 0.116) in exclusively patients with low‐stage disease (FIGO I‐II; Fig. S3).

Moreover, we quantified sMET among primary surgery and platinum‐based chemotherapy, reflected by four longitudinal follow‐up samples, obtained (a) within 1 week after primary surgery (*n* = 56), (b) before the onset of platinum‐based chemotherapy (*n* = 67), (c) after the first three cycles of chemotherapy (*n* = 56) and (d) after the completion of chemotherapy (*n* = 68; Fig. 1). After surgery, we observed a moderate increase in the median sMET level compared with primary diagnosis [estimated difference (ED) = 75.3 ng·mL^−1^, 95% CI: 6.7–143.9, *P* = 0.04] followed by a stabilization to baseline level at the onset of platinum‐based chemotherapy. Interestingly, after the first three cycles of chemotherapy, the median sMET level again transiently increased compared with primary diagnosis level, this time stronger than in the postsurgery samples (ED = 125.6 ng·mL^−1^, 95% CI: 57.6–190.9.1, *P* < 0.001; Fig. 1).

Conclusively, baseline levels of sMET at primary diagnosis of ovarian cancer do not differ from that of healthy controls but are transiently elevated in response to primary debulking surgery and platinum‐based chemotherapy.

### 3.2 Association of sMET serum level with clinicopathological parameters of ovarian cancer and CA125

**Fig. 2:**
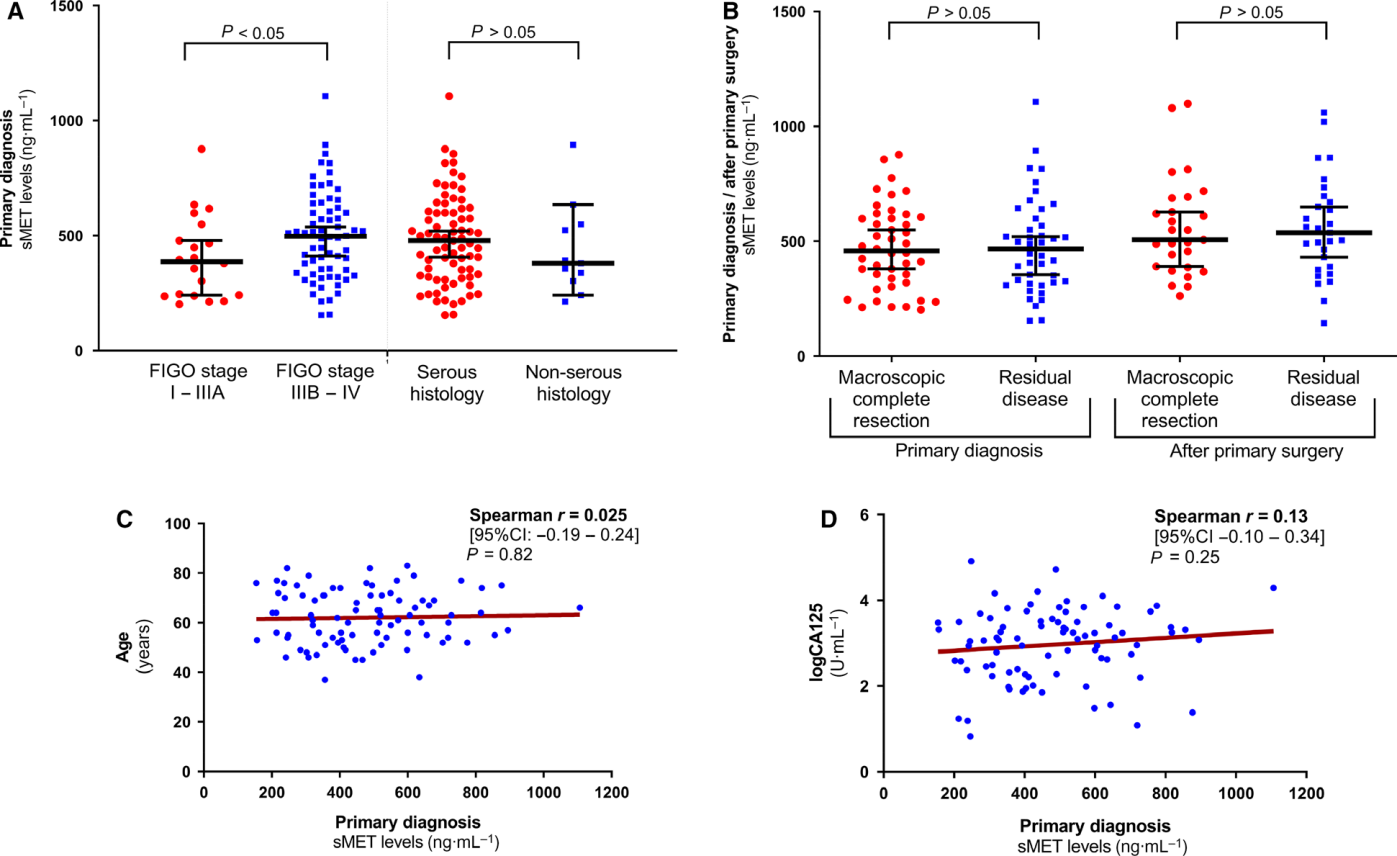
Association of sMET levels with clinicopathological data of ovarian cancer patients. Scatter plots comparing sMET levels between (A) FIGOI‐IIIA *vs*. FIGO IIIB‐IV ovarian cancer (*n*_total_ = 86) and serous histology *vs*. nonserous histology (*n*_total_ = 86) (B) patients with and without residual tumour left after primary debulking at primary diagnosis (*n*_total_ = 86) or after surgery (*n*_total_ = 56). The black horizontal lines indicate the median sMET level in each group, with error bars showing the 95% confidence interval (CI). *P*‐values, according to the nonparametric two‐tailed Mann–Whitney *U*‐test for independent samples, are indicated (C + D). Spearman correlation analysis between sMET levels and (C) age (*n* = 86) or (D) serum CA125 levels (*n* = 84 patients with matching CA125 values at primary diagnosis) with simple linear regression is shown.

**Fig. 3:**
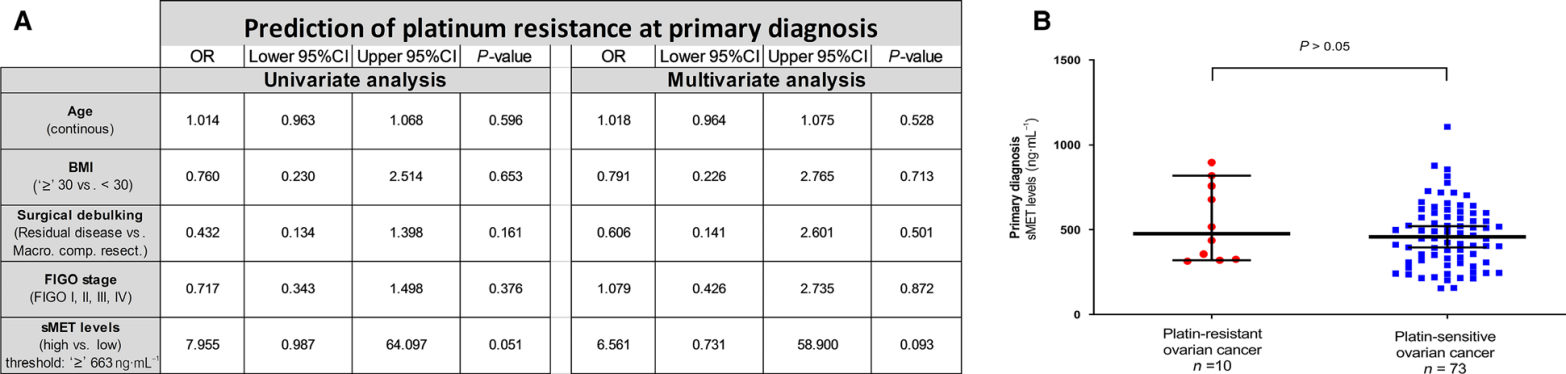
Prognostic relevance of sMET at primary diagnosis to predict primary platinum resistance. (A) Results are shown from univariate and multivariate generalized linear model analyses to predict platinum resistance including odds ratio (OR), 95% CIs. The cut‐off (663 ng·mL^−1^, *P* = 0.247, $n_{\left(>\;663\;\text{ng}\cdot {\text{mL}}^{‐1}\right)}$ = 13, $n_{\left(<\;663\;\text{ng}\cdot {\text{mL}}^{‐1}\right)}$ = 70) was determined as described in the Patients and methods section. (B) Scatter plot comparing sMET levels between primary platinum‐resistant ovarian cancer (*n* = 10) *vs*. primary platinum‐sensitive ovarian cancer (*n* = 73). The black horizontal lines indicate the median sMET level in each group, with error bars showing the 95% confidence interval (CI). *P*‐value according to the nonparametric two‐tailed Mann–Whitney *U*‐test for independent samples.

We correlated sMET levels at primary diagnosis of ovarian cancer with the patients' clinicopathological data. Higher levels of sMET correlated with advanced disease, indicated by a FIGO stage IIIB or IV (ED = 100.2 ng·mL^−1^, 95% CI: 5.9–191.4, *P* < 0.04; Fig. 2A). There was no association between sMET level and postoperative residual tumour burden left after primary debulking surgery, neither at primary diagnosis nor after surgery (Fig. 2B). There was no correlation between sMET levels in serum to either the patients' age (*r* = 0.025, 95% CI: −0.19 to 0.24, *P* = 0.82; Fig. 2C) or the histologic subtype (ED between serous vs. nonserous = 51.1 ng·mL^−1^, 95% CI: −178.0 to 71.2, *P* = 0.42; Fig. 2A). Interestingly, there was also virtually no correlation between sMET and serum CA125 at primary diagnosis (*r* = 0.13, 95% CI: −0.10 to 0.34, *P* = 0.25; Fig. 2D). Although primary platinum resistance was observed in only 10 patients with ovarian cancer, we performed an exploratory analysis of whether sMET levels at primary diagnosis could predict platinum resistance. There was no difference in the sMET levels between primary platinum‐resistant and platinum‐sensitive ovarian cancers (ED: −72.21 ng·mL^−1^, 95% CI: −219.8 to 82.9, *P* = 0.36) and no predictive information in the univariate or multivariate general linear model analysis (Fig. 3).

To conclude, sMET is mostly unrelated to common clinicopathological parameters, including CA125, and is only elevated in ovarian cancer patients with a high FIGO stage.

### 3.3 Prognostic relevance of sMET at primary diagnosis of ovarian cancer

**Fig. 4:**
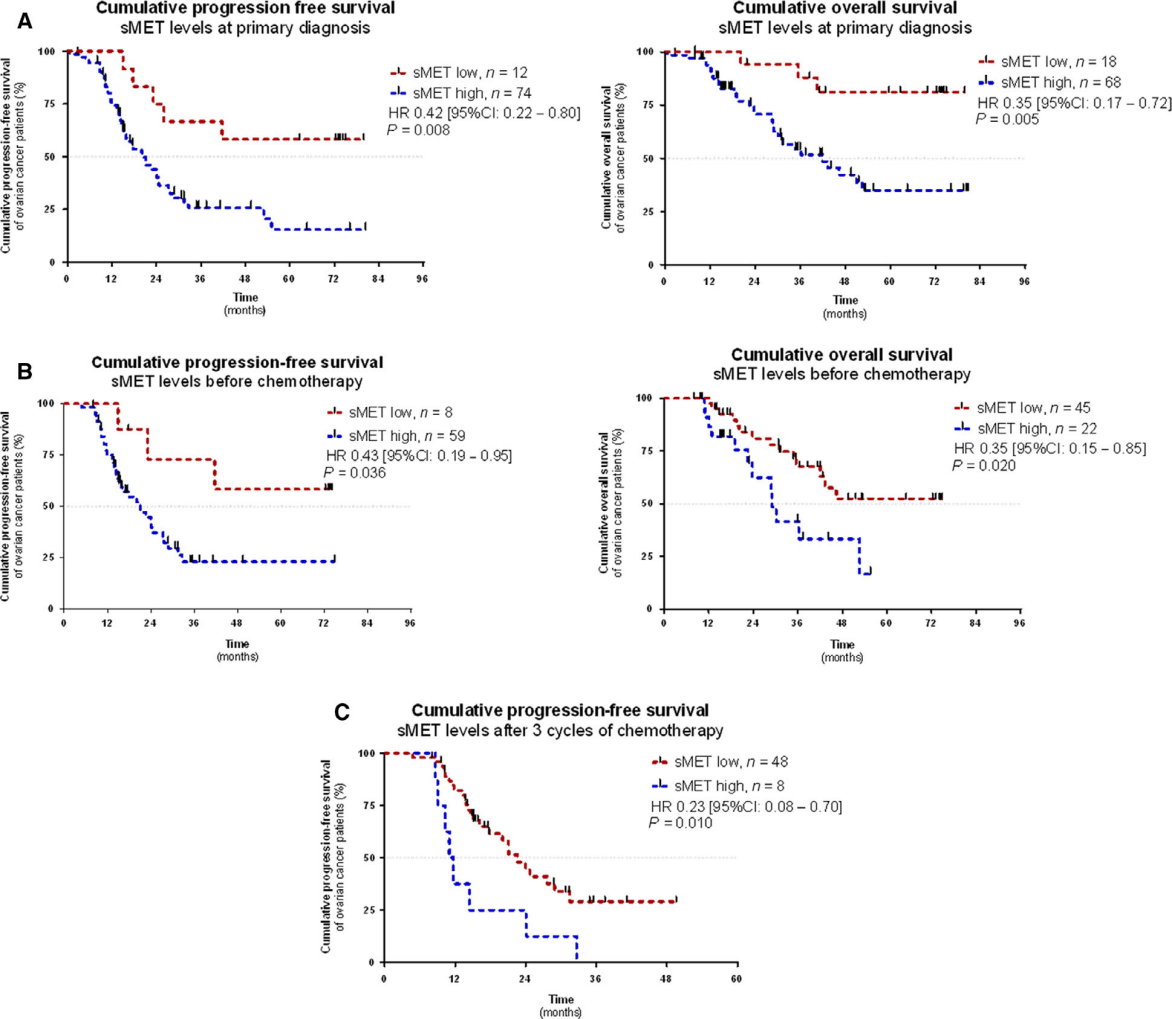
Prognostic relevance of sMET at primary diagnosis and in the course of platinum‐based chemotherapy. Kaplan–Meier analysis comparing progression‐free survival (PFS) and overall survival (OS) of patients with a high sMET level *vs*. patients with a low sMET level (A) at primary diagnosis (*n*_total_ = 86) (B) before the onset of platinum‐based chemotherapy (*n*_total_ = 67) and (C) PFS after the first three cycles of platinum‐based chemotherapy (*n*_total_ = 56). *P*‐values (log‐rank, Mantel–Cox) and hazard ratio (HR) (Mantel–Haenszel) were calculated as described in the Patients and methods section. The following cut‐offs were used as follows: primary diagnosis (PFS) = 246 ng·mL^−1^, primary diagnosis (OS) = 308.2 ng·mL^−1^, before chemotherapy (PFS) 267.7 ng·mL^−1^, before chemotherapy (OS) 567.1 ng·mL^−1^ and after three cycles of chemotherapy (PFS) = 792.8 ng·mL^−1^. Patient grouped into low sMET *vs*. high sMET as indicated.

Prognostic relevance was assessed by categorizing patients into either a sMET low group or a sMET high group using specific cut‐offs (Fig. S1). In the high sMET group, the percentage of patients with advanced disease (FIGOIII + IV) was comparatively increased and CA125 values were higher in this group compared with the sMET low group. There was no clear difference in histologic subtype and residual tumour load between patients in the two groups (Table S1). We subsequently performed univariate cox proportional hazards model analysis (univariate analysis) with low sMET *vs*. high sMET patients at primary diagnosis. A higher sMET level indicated reduced PFS (HR = 0.303, 95% CI: 0.118–0.777, *P* = 0.013) and reduced OS (HR = 0.206, 95% CI: 0.063–0.679, *P* = 0.009; Fig. S4). Kaplan–Meier analysis and the log‐rank test were additionally performed. Accordingly, patients with a high level of sMET had shorter PFS (HR = 0.42, 95% CI: 0.22–0.80, *P* = 0.008) and shorter OS (HR = 0.35, 95% CI: 0.17–0.72, *P* = 0.005; Fig. 4A).

We subsequently performed multivariate Cox proportional hazards regression model analysis (multivariate analysis) with PFS or OS as selected outcome variables, including sMET levels and established risk factors of ovarian cancer, that is age, body mass index (BMI), residual tumour load after primary surgical debulking and FIGO stage. We confirmed that an elevated sMET level was an independent predictor of shorter PFS (HR = 0.354, 95% CI: 0.130–0.968, *P* = 0.043) and shorter OS (HR = 0.217, 95% CI: 0.064–0.734, *P* = 0.014; Fig. S5).

### 3.4 Prognostic relevance of sMET at the individual longitudinal time points

We further analysed prognostic relevance of sMET in the longitudinal follow‐up samples, independently from each other, by disregarding the individual time‐dependent course. According to the univariate analysis, an elevated sMET level before onset of chemotherapy indicated shorter PFS (HR = 0.293, 95% CI: 0.087–0.982, *P* = 0.047) and OS (HR = 0.415, 95% CI: 0.190–0.904, *P* = 0.027; Fig. S4). Moreover, increased sMET after three cycles of platinum‐based chemotherapy also indicated shorter PFS (HR = 0.362, 95% CI: 0.163–0.808, *P* = 0.013). Similar results were observed by Kaplan–Meier analysis and the log‐rank test (Fig. 4B,C).

According to the multivariate analysis, an elevated sMET level before the onset of chemotherapy was an independent predictor for shorter OS (HR = 0.384, 95% CI: 0.158–0.932, *P* = 0.034). Moreover, the time points after surgery and after the first three cycles of platinum‐based chemotherapy, at which a transient increase in the median sMET level was observed, were prognostically most informative and constituted an independent predictor for shorter PFS (after surgery: HR = 0.319, 95% CI: 0.121–0.842, *P* = 0.021, after three cycles of chemotherapy: HR = 0.245, 95% CI: 0.100–0.602, *P* = 0.002; Fig. S5). Other individual time points did not offer independent prognostic information for PFS and OS (Fig. S5).

Taken together, sMET allows independent prognostic stratification of ovarian cancer patients among specific longitudinal follow‐up samples, with sampling after three cycles of platinum‐based chemotherapy as the most prognostically informative point.

### 3.5 Prognostic relevance of patients' individual sMET dynamics

**Fig. 5:**
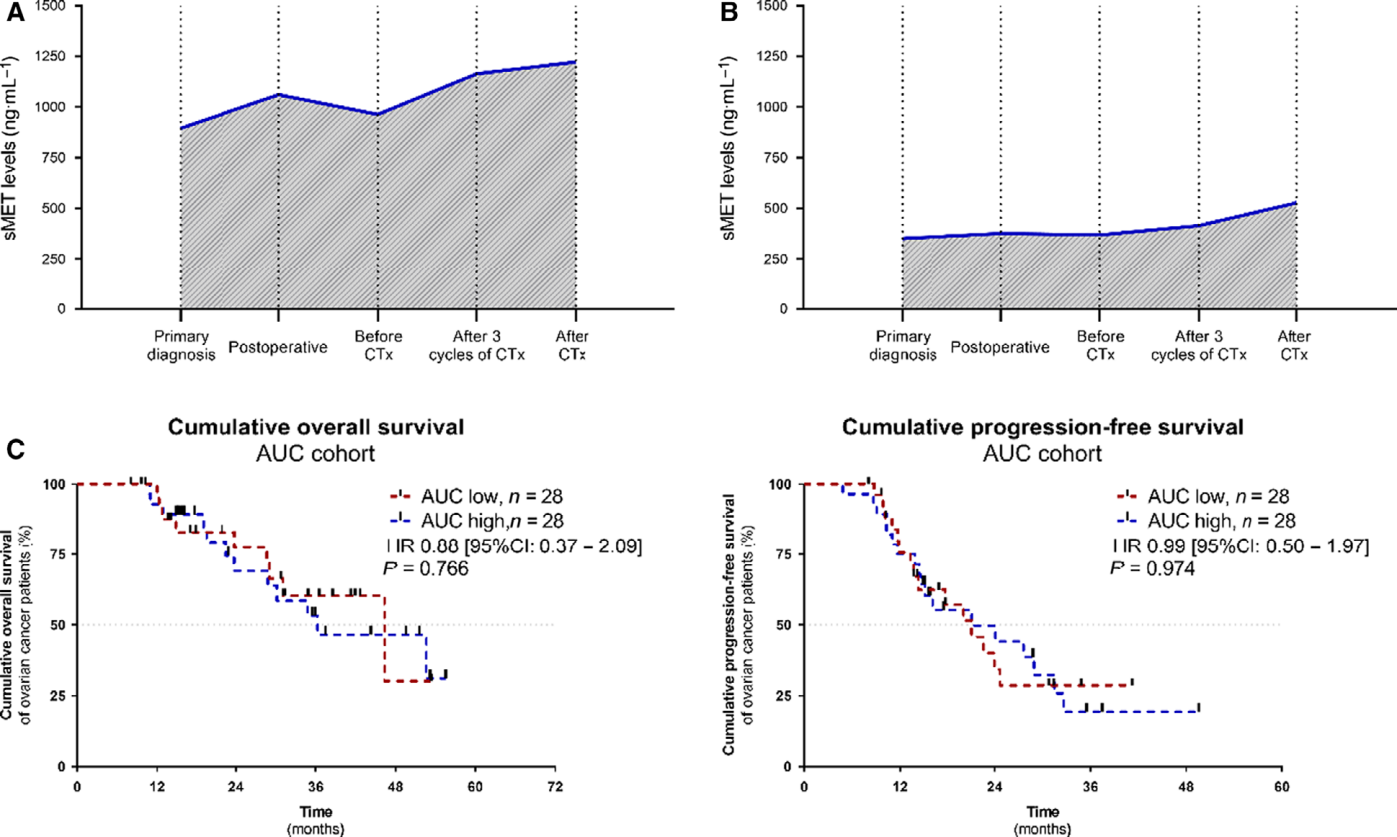
Prognostic relevance of the patient individual sMET dynamics. Patient's dynamic curves showing the progression of sMET levels between primary diagnosis and the completion of chemotherapy. (A) Example of an individual patient with a high AUC and (B) example of an individual patient with a low AUC. (C) Kaplan–Meier analysis comparing progression‐free survival (PFS) and overall survival (OS) of ovarian cancer patients (*n*_total_ = 56) in the AUC high group *vs*. AUC low group. The median was used as cut‐off. *P*‐values (log‐rank, Mantel–Cox) and hazard ratio (HR) (Mantel–Haenszel) were calculated as described in the Patients and methods section.

For 56/86 patients, a set of five longitudinal serum samples throughout primary treatment was available. In addition to the previous analyses, we then inquired whether the dynamics of sMET in each individual patient (*n* = 56) is of prognostic relevance. Assuming a linear and continuous change in sMET levels between the investigated samples, we plotted a dynamic curve for each patient using all available sMET levels across treatment. By setting the different time points of sample analysis as categorical variables, we calculated patient‐specific AUCs, each of them reflecting the individual sMET dynamics in the course treatment. All patients were stratified into the ‘AUC high’ group or into the ‘AUC low’ group (Fig. 5A,B). In the univariate analysis, high sMET levels indicated shorter PFS (HR = 0.365, 95% CI: 0.183–0.729, *P* = 0.004). This was also reflected in the multivariate analysis (HR = 0.326, 95% CI: 0.140–0.755, *P* = 0.009). However, OS was not significantly different for these distinct patient groups and analyses (Fig. 5C and Figs S4 and S5). Therefore, the individual dynamics of sMET is an independent prognostic marker for PFS but not OS.

### 3.6 sMET level in relapsed ovarian cancer

**Fig. 6:**
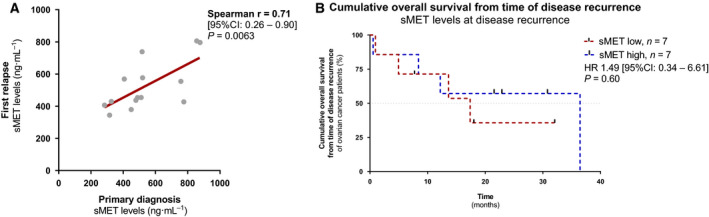
sMET level in recurrent ovarian cancer. (A) Spearman correlation analysis between sMET levels at primary diagnosis *vs*. at disease relapse (*n* = 14) with linear regression shown (red line). (B) Kaplan–Meier analysis comparing progression‐free survival (PFS) and overall survival (OS) of patients with a high sMET serum level *vs*. patients with a low sMET serum level at the time of first relapse (*n*_total_ = 14). The median was used as cut‐off. *P*‐values (log‐rank, Mantel–Cox) and hazard ratio (HR) (Mantel–Haenszel) were calculated as described in the Patients and methods section.

In 14/86 cases, we could obtain matched serum samples at primary diagnosis *vs*. disease relapse. We observed a strong correlation between sMET levels at primary diagnosis and at the time of relapse (*r* = 0.71, 95% CI: 0.26–0.90, *P* = 0.0063; Fig. 6A). However, sMET levels at relapse were prognostically noninformative (Fig. 6B).

## 4 Discussion

In the present study, we investigated the clinical relevance of sMET as a potential blood‐based biomarker for ovarian cancer patients. Although the median sMET level at primary diagnosis of ovarian cancer did not differ to that of healthy controls, we show that sMET levels at primary diagnosis and in the course of platinum‐based chemotherapy were an independent prognostic biomarker.

Ectodomain shedding of cMET occurs under physiological conditions and in malignant tissue. It is thought to be the origin of sMET, which is stably detectable in serum, plasma and urine of diseased and healthy individuals [27, 30, 31, 32]. Ectodomain shedding is mainly attributed to members of the ADAM (a disintegrin and metalloprotease) family of proteases and to γ‐secretase. ADAM10 and ADAM17 are well‐characterized and have functionally been associated with shedding of cMET and other surface proteins, such as E‐cadherin, EGFR or NGFR [26, 41, 42, 43]. Shedding of cMET is a highly regulated process and can be stimulated by other pathways, such as EGFR signalling or integrin ligation [44].

Since no difference was found between the median sMET levels in ovarian cancer patients *vs*. healthy controls, there was poor diagnostic discrimination of these two groups (AUC = 0.56). This has also been shown in previous studies on lung cancer and multiple myeloma which also reported no difference in sMET levels in the respective total patient cohorts compared with healthy controls [31, 34]. In our study, there was also no association between sMET levels shortly after surgical debulking (within 7 days) and residual tumour load. This suggests that there is a poor correlation between a patient's individual tumour load and sMET level and could also explain why there was no significant difference between the median sMET levels at disease relapse and at primary diagnosis. A similar observation has also previously been reported in a study on lung cancer [31]. Here, an increase in plasma sMET was exclusively restricted to patients with cMET overexpression in the primary tumour (50% of cases), and, importantly, there was a poor correlation between sMET level and tumour size in patients with cMET‐overexpressing tumours [31]. This supports the notion that sMET could be a blood‐based surrogate for cMET overexpression in the primary tumour [28, 31]. However, there is considerable range in cMET expression, ranging from 10.9% to 76.6% [21], likely due to the fact that there is still no clinically validated methodological consensus for defining cMet overexpression. Moreover, it is generally difficult to determine which biological threshold of cMET shedding is necessary to measurably increase sMET levels. However, it cannot be ruled out that there is also a fraction of cMET‐overexpressing tumours that do not trigger a strengthened release of sMET into the circulation. In this regard, direct comparison between blood‐based sMET level and primary tumour based cMET expression in our patients was beyond the scope of the present study and we focussed on sMET as a discrete blood‐based biomarker. We conclude that measuring sMET at primary diagnosis, at least without characterization of cMET status in the primary tumour, is neither informative for diagnostic screening nor informative for accurately assessing a patient's tumour load in ovarian cancer.

Interestingly, there is a transient increase in sMET levels (a) within 7 days postsurgery and (b) after the first three cycles of chemotherapy. It is likely that physical traumata, conferred by surgery, or toxicity conferred by chemotherapy, may stimulate cMET shedding and its release into circulation. This is consistent with the fact that HGF and cMET are upregulated after tissue damage, such as kidney, liver or heart injury, suggesting that the HGF/cMET pathway is generally involved in tissue damage response and tissue regeneration [45, 46, 47]. Moreover, a report on a mouse model of 3,5‐diethoxycarbonyl‐1,4‐dihydroxycollidine (DDC)‐induced hepatobiliary obstruction clearly indicated that ADAM10/17‐mediated cMET shedding and sMET levels correlated with the degree of liver damage [41].

We observed a strong and independent prognostic relevance of this marker at primary diagnosis, which is in accordance with the already known prognostic relevance of cMET overexpression in primary ovarian cancer tissue [23]. The fact that sMET at primary diagnosis has a strong prognostic impact, while sMET levels at this time‐point did not significantly differ compared with healthy controls, might seem counterintuitive at the first sight. However, prognostic relevance of a given (blood‐based) discrete biomarker at primary diagnosis is likely but not necessarily associated with elevated levels of this biomarker at primary diagnosis *vs*. healthy controls, since only the interpatient variability is of importance for this kind of analysis.

Nonetheless, our study offers new insights into the potential clinical application of sMET and its use as a discrete and blood‐based readout for prognostic stratification in ovarian cancer patients, without the need to characterize cMET expression in the primary tumour. In this regard, high sMET levels at primary diagnosis identify patients with a more aggressive disease, that is those with a high risk of recurrence and higher mortality. Interestingly, there was virtually no correlation between sMET and serum CA‐125, indicating that sMET could be complimentary to CA‐125 as a biomarker for ovarian cancer patients.

Our blood‐sampling strategy allows sMET analysis at primary diagnosis and along longitudinal follow‐up samples in the course of primary treatment. We further identified the independent prognostic relevance of sMET after the first three cycles of platinum‐based chemotherapy and propose that sMET could be a potential blood‐based biomarker for monitoring the clinical benefit of platinum‐based chemotherapy in addition to CA125. This is in line with recent work on advanced lung cancer, for which sMET was suggested to act as a dynamic monitoring marker of EGFR‐TKI treatment [36]. There was no difference in sMET levels of patients with primary platinum‐resistant and platinum‐sensitive ovarian cancer. Furthermore, sMET did not offer predictive information for primary platinum resistance. The functional role of the HGF/cMET axis in platinum resistance remains controversial [48, 49].

Our general conclusion that higher sMET levels at the reported individual time points identify ovarian cancer patients with poor prognosis could be explained by the observation that these patients bear tumours with increased cMET shedding and tumour cells with a more aggressive behaviour. This hypothesis is supported by a previous observation, describing a positive correlation of cMET ectodomain shedding and the malignant potential of tumour cells *in vitro* [27]. However, increased sMET levels in ovarian cancer patients with an unfavourable prognosis do not necessarily allow the conclusion that these tumours have increased HGF/cMET signalling. It has also been reported that sMET can act as a decoy receptor, which binds and sequesters HGF, resulting in decreased HGF/cMET signalling [50, 51].

## 5 Conclusion

This is the first study suggesting sMET can be used as a blood‐based and independent prognostic biomarker for ovarian cancer patients at primary diagnosis and in the course of platinum‐based chemotherapy. Since sMET is an easily detectable serum parameter, it could be implemented into standard diagnostic procedures as a complementary tumour marker for individualized prognosis stratification in ovarian cancer and for monitoring treatment response. Ovarian cancer patients with high risk of recurrence, as identified by sMET, may benefit from targeted therapy regimes, such as immunotherapy or PARPi. In addition, determining sMET levels at primary diagnosis may predict response to cMET‐targeted therapy, which has previously only been tested in ovarian cancer patients without prior cMET analysis [24, 25].

## Conflict of interest

The authors declare no conflict of interest.

## Author contributions

DMK, JDK, MG, PW and TL made substantial contributions to the conception and design of the study. DMK and JDK contributed to the experimental work or to the acquisition of data and to the analysis/interpretation of the results. JDK and DMK were involved in drafting the manuscript, creating figures and revising the manuscript. DMK initiated the study. All authors read and approved the manuscript in its final version.

## Supporting information

**Fig. S1.** Numerical and graphical summary of cut off determination (A) Determination of fixed sMET cut‐offs for Kaplan‐Meier analysis, categorizing patients into a sMET high and sMET low group by maximally selected log‐rank statistics using conditional Monte‐Carlo as p value approximation. Ovarian cancer at primary diagnosis (n = 86), after primary surgery (n = 56), before chemotherapy (n = 67), after three cycles of chemotherapy (n = 56), after completion of chemotherapy (n = 68) (B) The graphical representation of the cut off identification is shown for the primary diagnosis for sMET (OS).

**Fig. S2.** Prognostic relevance of an arbitrarily selected cut off. Kaplan Meier analysis comparing (A) progression‐free survival (PFS) and (B) overall survival (OS) of sMET low *vs*. sMET high patients at primary diagnosis (n_total_ = 86). P‐values (log‐rank, Mantel–Cox) and hazard ratio (HR, Mantel Haenszel) were calculated as described in the Patients and methods section. The cut off was selected as 330ng/mL to be close to statistically determined cut offs (PFS: 246.0 ng/mL and OS: 308.2 ng/mL, determined by maximally selected log‐rank statistics).

**Fig. S3.** Diagnostic capacity of sMET at primary diagnosis. For different thresholds of sMET level, the true positivity rate (sensitivity) was plotted against the false positivity rate (100%‐specifity) in order to analyze the diagnostic ability of sMET to distinguish between ovarian cancer patients and healthy controls (n = 85) in (A) the total cohort comprising all FIGO stages (n = 86) and (B) ovarian cancer patients with low stage disease (FIGO I‐II, n = 13). The area under the curve (AUC) and the 95% confidence interval (CI) are indicated.

**Fig. S4.** Univariate prognostic relevance of sMET level. (A) Results from univariate cox proportional hazards regression model analysis at all investigated time points, including hazard ratio (HR) and 95% confidence interval (CI). (B) Graphical presentation of HRs with regard to progression‐free survival (PFS) and overall survival (OS). The following ovarian cancer patients were analyzed: at primary diagnosis (n = 86), after surgery (n = 56), before chemotherapy (n = 67), after 3 cycles of chemotherapy (n = 56) and after chemotherapy (n = 68). Cut offs for AUC cohort (n = 56) is the median and the other cut offs are as shown in Supplementary Figure 1.

**Fig. S5.** Multivariate prognostic relevance of sMET level. (A) Results from multivariate cox proportional hazards regression model analysis at all investigated time points of longitudinal blood drawing, including hazard ratio (HR) and 95% confidence interval (CI). (B) Graphical presentation of HRs with regard to progression‐free survival (PFS) and overall survival (OS). The following ovarian cancer patients were analyzed: at primary diagnosis (n = 86), after surgery (n = 56), before chemotherapy (n = 67), after 3 cycles of chemotherapy (n = 56) and after chemotherapy (n = 68). Cut offs for AUC cohort (n = 56) is the median and the other cut offs are as shown in Supplementary Figure 1.

**Table S1.** Patient characteristics at primary diagnosis according to sMET levels

## Data Availability

All relevant data and descriptions of statistical workflows are contained in the manuscript. Raw data of ELISA‐based sMET detection can be made available from the author (Daniel.m.klotz@ukdd.de) upon reasonable request.
