# Supplementary figures and images for: The levels of soluble cMET ectodomain in the blood of patients with ovarian cancer are an independent prognostic biomarker
Source: Mol Oncol. 2021 Apr 7;15(9):2491–503. doi: 10.1002/1878-0261.12939 (PMC8410524; doi:10.1002/1878-0261.12939)

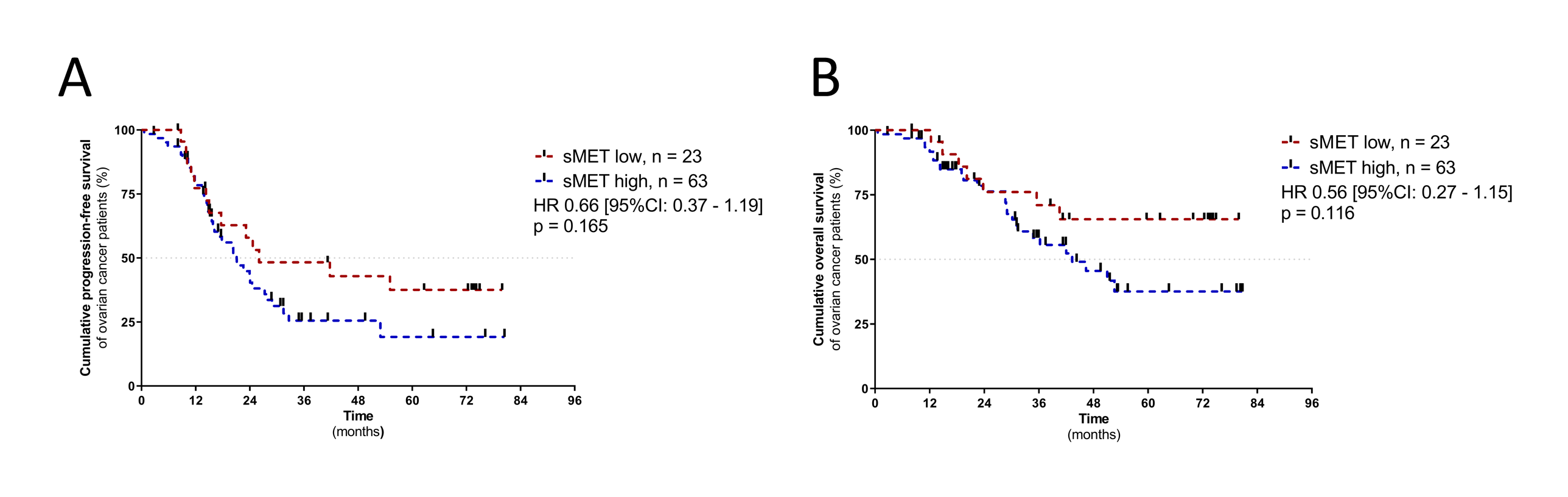

Supplement: Supplementary file 2 — Fig. S2. Prognostic relevance of an arbitrarily selected cut off. Kaplan Meier analysis comparing (A) progression‐free survival (PFS) and (B) overall survival (OS) of sMET low vs. sMET high patients at primary diagnosis (ntotal = 86). P‐values (log‐rank, Mantel–Cox) and hazard ratio (HR, Mantel Haenszel) were calculated as described in the Patients and methods section. The cut off was selected as 330ng/mL to be close to statistically determined cut offs (PFS: 246.0 ng/mL and OS: 308.2 ng/mL, determined by maximally selected log‐rank statistics). [file MOL2-15-2491-s003.tif]
