# Supplementary material for: The levels of soluble cMET ectodomain in the blood of patients with ovarian cancer are an independent prognostic biomarker
Source: Mol Oncol. 2021 Apr 7;15(9):2491–503. doi: 10.1002/1878-0261.12939 (PMC8410524; doi:10.1002/1878-0261.12939)
Supplement: Supplementary file 6 — Table S1. Patient characteristics at primary diagnosis according to sMET levels [file MOL2-15-2491-s005.docx]

**Supplementary Table 1**

| **Patients characteristics at primary diagnosis according to sMET levels** | | |
| --- | --- | --- |
| **Patient subgroup** | **Low sMET** | **High sMET** |
| N | 18 | 68 |
|  |  |  |
| **Cut off** | below 308.2 ng/mL | above 308.2 ng/mL |
|  |  |  |
| **Age** | median 64 years (46 - 82 years) | median 61 years (37 - 83 years) |
|  |  |  |
| **BMI** | median 25.2 ( 21.8 - 36) | median 26.8 ( 19.6 - 39.7) |
|  |  |  |
| **FIGO** |  |  |
| I - II | 5 (27.8%) | 8 (11.8%) |
| III - IV | 13 (72.2%) | 60 (88.2%) |
|  |  |  |
| **Histologic type** | |  |
| serous | 15 (83.3%) | 60 (88.2%) |
| other | 3 (16.7%) | 8 (11.8%) |
|  |  |  |
| **Recurrence** |  |  |
| PFS | median 41 months (8 - 80 months) | median 16 months (1 - 81 months) |
| no relapse | 9 (50%) | 25 (36.8%) |
| relapse | 9 (50%) | 43 (63.2%) |
|  |  |  |
| **Survival** |  |  |
| OS | median 61 months (8 - 80 months) | median 29 months (1 - 81 months) |
| alive | 15 (83.3%) | 37 (54.4%) |
| dead | 3 (16.7%) | 31 (45.6%) |
|  |  |  |
| **Residual Tumor** | |  |
| macroscopic complete resection | 10 (55.6%) | 32 (47.1%) |
| any residual tumor | 8 (44.4%) | 36 (52.9%) |
|  |  |  |
| **CA125** [U/mL] | median 379 U/mL (7 - 4882 U/mL) | median 903 U/mL(12 - 6055 U/mL) |
